# Multifunctional Medical Recovery and Monitoring System for the Human Lower Limbs

**DOI:** 10.3390/s19225042

**Published:** 2019-11-19

**Authors:** Adriana Comanescu, Ileana Dugaesescu, Doru Boblea, Liviu Ungureanu

**Affiliations:** Mechanisms and Robots Theory Department, Faculty of Industrial Engineering and Robotics, University Politehnica of Bucharest, Bucharest 060042, Romania; ileana.d1@gmail.com (I.D.); doruboblea@gmail.com (D.B.); ungureanu.liviu.marian@gmail.com (L.U.)

**Keywords:** mechanism with three degrees of mobility, direct structural model, inverse structural model, active pair, modular groups connection, kinematic modelling, dynamic modelling, sensor for angular amplitude

## Abstract

In order to develop multifunctional medical recovery and monitoring equipment for the human lower limb, a new original mechanical structure with three degrees mobility has been created for the leg sagittal model. This mechanism is integrated in the equipment and includes elements that have similar functions to the different anatomic parts (femur, median part), leg, and foot. The independent relative rotation motion between the previously mentioned anatomic parts is ensured. The femur may have an oscillation rotation of about 100° relative to the trunk. The median part (leg) alternatively rotates 150° relative to the superior segment. The lower part (foot) is initially placed at 90° relative to the median part and may have an alternative rotation of 25°. Depending on a patient’s medical needs and their recovery progress, device sensors provide varying angular amplitude of different segments of the human limb. Moreover, the mechanism may actuate either anatomic leg segment, two parts, or all of them.

## 1. Introduction

**Figure 1:**
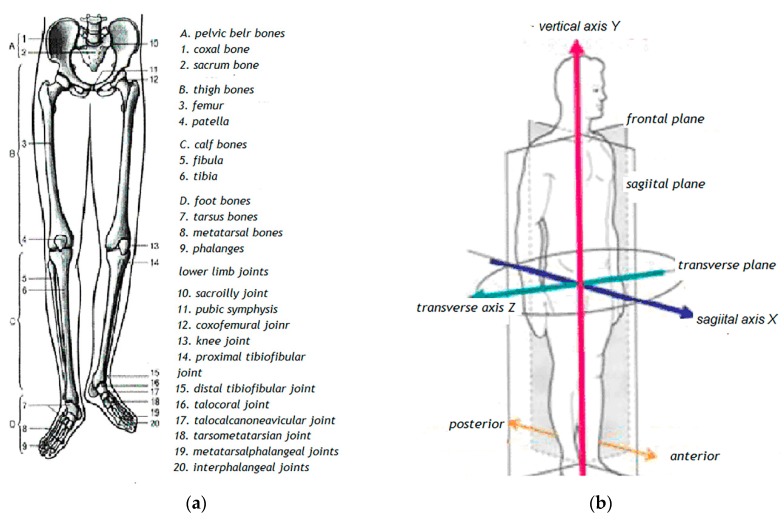
Lower human limb: (**a**) Main parts; (**b**) sections and axes.

The lower human limb (Figure 1) allows motions relative to its main parts. A mechanism with three degrees of mobility has been used to created dedicated medical equipment for the multifunctional rehabilitation and monitoring of the lower limb [1,2]. Some of its three links receive the roles of the main parts of the human leg main parts, i.e., the thigh, leg, and foot regions. The system makes it possible to alternatively rotate each part, two parts, or all of them at the same time. In accordance to the standard movement characteristics of the lower limb, the relative rotation amplitudes are ensured. Thus, the thigh may oscillate about 100° relative to the trunk, the leg about 100° relative to the superior part, and the foot about 90° relative to the leg.

Some equipment is used to recover lower limb joint function. This type of medical device is dedicated to active limb rehabilitation—its ankle joint for flexion, extension, pronation, and supination movements [3]. The educational device and mini stepper share the same function [4,5]. The mini talus device is used to enhance ankle joint stability [6]. To recover the knee joint function after a medical intervention, the device is also recommended [7]. Other equipment [8] can also be used to improve and rehabilitee the lower limb muscles and tendons.

In this paper, the three degrees of mobility mechanism are presented as new equipment, bringing together many functional aspects that have been previously mentioned in other studies [9,10].

In the following sections, we present theoretical research and fundamental principles that have been adopted from robotics and the theory of mechanisms, which underlie the construction and use of this mechanism with three degrees of mobility. In order to be integrated into medical equipment, the construction must be based on closed contours. The serial model mentioned by Glowinski and Krzyzynski is inadequate for the proposed purpose.

In Section 2, the structural considerations regarding this mechanism are presented. These use different phases of work pertaining to the direct and inverse structural models, which include modules with varying degrees of mobility.

In the following two sections, the kinematic and dynamic characteristics of the mechanism are presented for the exclusive recovery of the coxofemoral joint. Modeling algorithms use the classical calculation modules in mechanism theory. Using them in any programming language with the relationships allows for the reproduction of the results for other input data.

Similarly, research is developed in order to model the mechanism for exclusive knee and ankle joint training. The diagrams in each section demonstrate the correctness of the relationships, which are derived from the classical theory of mechanisms in the modeling algorithms and the utility of the mechanism for the designed equipment.

## 2. The Structural Model of the Human Lower Limb

**Figure 2:**
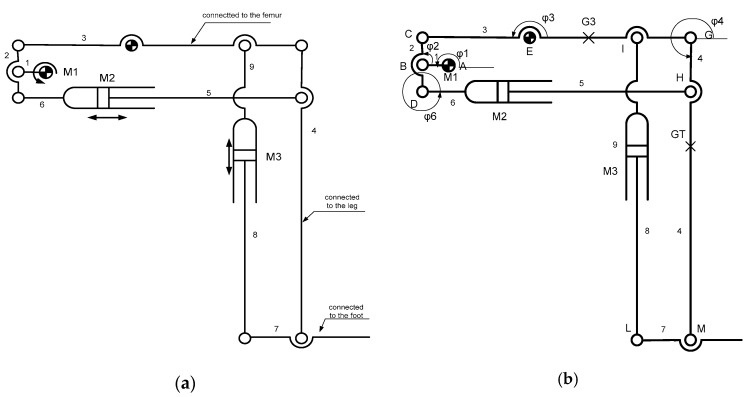
Mechanism with three degrees of mobility: (**a**) Connection to the lower limb; (**b**) positional characteristics of the mechanism with three degrees of mobility.

In the sagittal plane, the human lower limb model used for medical recovery and monitoring is represented by a new mechanism (Figure 2) with three degrees of mobility. This system [1,2] ensures independent movements from the coxofemural, knee joints, and ankle joints. The lower limb segments are placed as follows: the femur part on link 3, the median part on element 4, and the foot on link 7. The mechanism (Figure 2) has *m* = 9 mobile elements and *i*= 12 kinematic lower pairs. Each imposes two constraints in the relative motion of the adjacent links. The degree of mobility denoted by *M* is given by *M* = 3*m* − 2*i* and *M* = 3. Thus, the system has three degrees of mobility and three independent parameters, which define the motion state. At the same time, it also defines its number of active pairs [2]. The active pairs (Figure 2a,b) are the following: *M*1 between the base and link 1; *M*2 between links 5 and 6; and *M*3 between links 8 and 9. The mechanism also has three independent contours (*N* = 3*)* verified by means of the classical formula (*N* = *i − m*).

**Figure 3:**
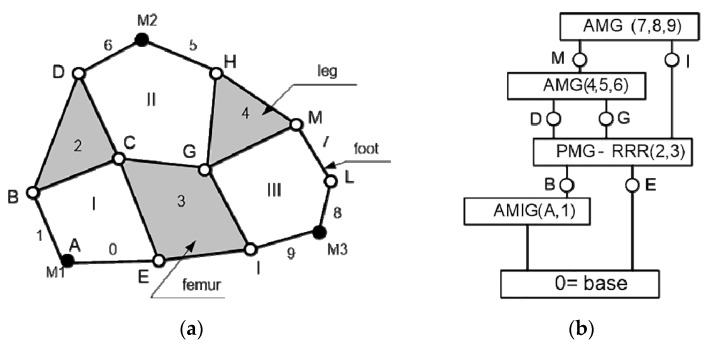
Mechanism with three degrees of mobility: (**a**) Direct structural model of the mechanism; (**b**) modular group connection.

The direct structural model of the mechanism is given in Figure 3a. It is a conventional design adopted by the mechanism theory in which the elements and links are represented by polygons with a number of peaks equal to the number of pairs. All lower pairs are similarly drawn. The active pairs are also marked.

During the function of the active pair placement, the modular group connection (Figure 3b) is achieved. According to the mechanism construction fundamental principle, each planar system is a connection of active modular groups (AMG) and/or passive modular groups (PMG). Some passive modular groups connect to Baranov trusses (i.e., systems with zero degrees of mobility and three degrees of freedom). These are presented in Table 1.

In Table 2, any active modular group with one degree of mobility is shown [2]. In the case of the already presented mechanism (Figure 2), the modular group connection (Figure 3b) includes three active groups with a single degree of mobility: the first one is the modular active initial group (AMIG) and the others are AMG (4, 5, 6) and AMG (7, 8, 9). Only one PMG (2, 3) is classically named dyad [2].

**Figure 4:**
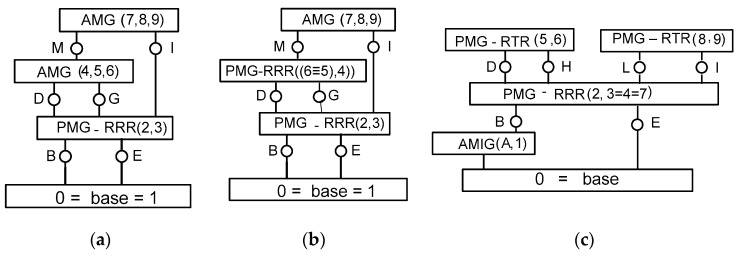
Mechanism with three degrees of mobility: (**a**) Modular configuration where *M*1 is blocked; (**b**) modular configuration maintaining the leg and the foot in the convenient fixed position direct showing the structural model of the mechanism; (**c**) modular groups connected for the alleged solidarity of the leg with the femur and the foot with the leg.

By using the modular group connection (Figure 3b), one may justify the various functional stages of the device mechanism. Consequently, the following aspects are distinct:

- It is necessary to use all active pairs to exclusively exercise the coxofemural joint. *M*1 ensures the motion of the femur relative to the trunk, when *M*2 and *M*3 maintain the relative fixed position of the leg and foot. The connection presented in Figure 3b is available and can be applied to estimate the characteristics of all active pairs.
- In order to exclusively move the knee joint, *M*1 is blocked in the desired position. At the same time, *M*3 ensures the position of the foot relative to the leg. *M*2 determines the motion of the knee. The modular configuration is given in Figure 4a. In its construction, it has a single modular passive group (Table 1) denoted by RRR (2, 3) and two active ones (Table 2) given by AMG–RTRR (4, 5, 6) and AMG–RTRR (7, 8, 9).
- To act only the talocrural joint according to the medical parameters maintaining in the convenient fixed position the leg and the foot, the active pairs *M*1 and *M*2 are used to place the human member segments in the desired positions and *M*3 to determine the movement of the foot. The modular configuration for this section is shown in Figure 4b. Two modular passive groups given by PMG–RRR (2, 3) and PMG–RRR ((6 ≡ 5), 4) and a single mono-mobile one marked AMG–RTRR (7, 8, 9) are relevant for the proposed purpose. In the following, there are various cases of using the mechanism for functional recovery of the lower human joints.

The structural solution for the lower human member includes mechanisms with a similar purpose than previous research [11,12,13,14].

## 3. Mechanism Kinematic Characteristics for the Exclusive Recovery of the Coxofemural Joint Function

The mechanism with its three active pairs is shown in Figure 2. *M*1 is an active rotation pair and is placed in A between the base and link 1. *M*2 and *M*3 are active prismatic pairs, respectively, and place between links 5 and 6 and links 8 and 9.

Taking into account the medium standard anatomic dimensions of the human lower limb, the geometrical constant parameters of the mechanism shown in Figure 2 are presented in Table 3. The links masses of the specific regions–femur, leg and foot are similarly selected.

**Figure 5:**
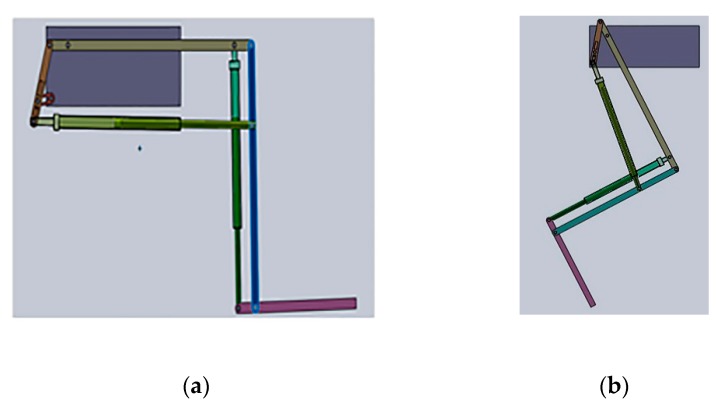
Exercises for the coxofemural joint: (**a**) femur initial position; (**b**) femur extreme position

In order to exclusively make the exercises for the coxofemural joint according to the medical parameters, as well as maintaining a fixed position at 90° of the leg relative to the femur and of the foot relative to the leg (Figure 5), it is necessary to use all active pairs (*M*1, *M*2, and *M*3).

By using the parameters mentioned in Figure 2 and the geometrical characteristics of the mechanism elements from Table 3, the algorithm for the positional modeling of the mechanism [15,16] is presented in Table 4. 

Virtually, this situation corresponds to the alleged solidarity of the leg with the femur and the foot with the leg. Thus, the new connection of modular groups is given in Figure 4c.

**Figure 6:**
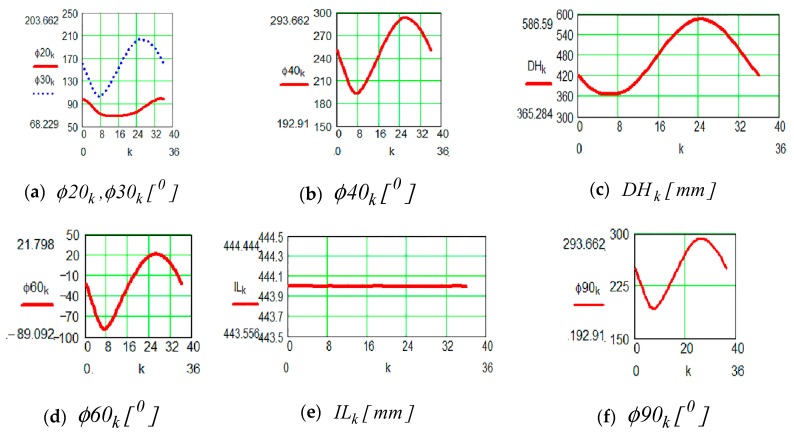
Modular group positional characteristics of the mechanism for the exclusive recovery of the coxofemoral joint function: (**a**) Dependent parameters of RRR (2, 3); (**b**) angular dependent parameter ϕ40 (Figure 3); (**c**) linear dependent parameter of RTR (6, 5); (**d**) angular dependent parameter of RTR (6, 5); (**e**) linear dependent parameter of RTR (9, 8); (**f**) angular dependent parameter of RTR (9, 8).

Following the modular group connection (Figure 4c) and the algorithm given in Table 4, the angles ϕ2 and ϕ3, which characterize the passive modular group PMG–RRR (2, 3), are firstly determined. Figure 6a shows their variation curves expressed in degrees by ϕ20 and ϕ30. In order to preserve the angular position between link 3 (femur) and link 4 (leg), the active pair (*M*2) must determine the ϕ40 angular position for link 4, as shown in Figure 6b.

Following the algorithm from Table 4, the DH linear parameter and the ϕ60 angular parameter respectively given in Figure 6c,d are emphasized. In this medical phase, the foot is placed at 90° relative to the leg. The algorithm gives the values for the IL(linear) and ϕ90 (angular parameters), which are shown in Figure 6e,f.

**Figure 7:**
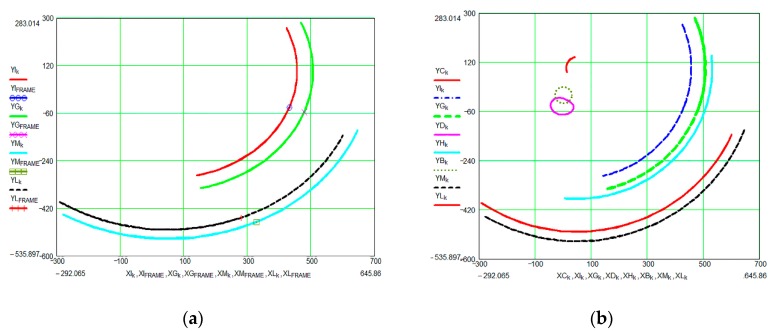
The mechanisms trajectory of various points in simulation: (**a**) marked initial positions; (**b**) significant points of mechanisms.

Figure 7 presents trajectories of various points for link connections, where the initial positions are also marked. The curves from Figure 7 demonstrate the constant values for the IL in comparison to the length of link 4.

## 4. Mechanism Dynamic Characteristics for the Coxofemoral Joint Function Recovery

All active pairs (*M*1, *M*2, and *M*3) are involved in exclusive exercises of the coxofemoral joint, according to the medical parameters. This is to maintain the leg’s fixed positions of 90° relative to the femur and foot relative to the leg (Figure 2b), respectively. The associated modular group connection of the mechanism is shown in Figure 3b. The dynamic characteristics of the system [2] according to this functional phase are determined using its geometrical and mass links characteristics from Figure 2b and Table 3, as well as the algorithm given in Table 4.

The modular group connection (Figure 3b) applies the direct structural model and dynamic modules of the following modular groups: AMG-RTRR, PMG-RRR, and AMIG specifically presented in [2].

**Figure 8:**
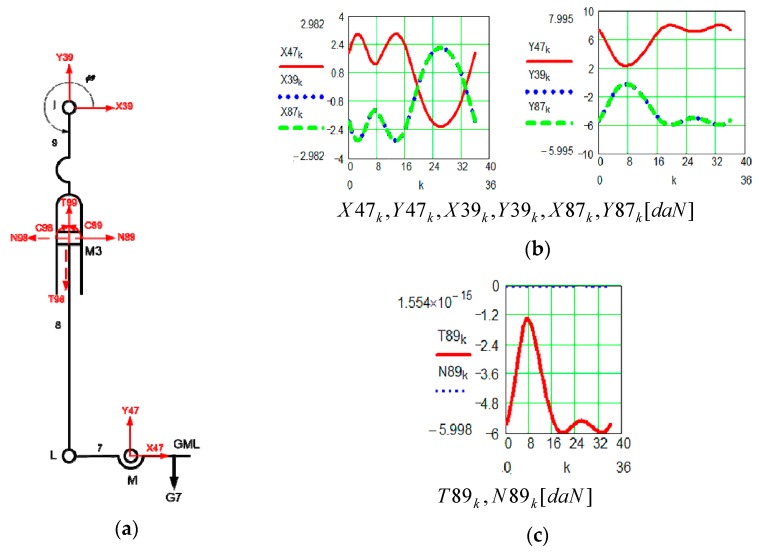
Active modular group RTRR (7, 8, 9): (**a**) reaction torque in each pair; (**b**) reaction forces variation in the rotation pairs; (**c**) reaction forces variation in the active prismatic pair.

Thus, for the RTRR (9, 8, 7) active modular group from Figure 8a, the reaction torque in each kinematic pair may be established, the algorithm being given in Table 5.

The reaction torques (*X47_k_, Y47_k_*) in pair *M*, (*X39_k_, Y39_k_*) in pair *I*, (*X87_k_ = −X78_k_*, *Y87_k_ = −Y78_k_*) in pair *L*, and *(N89_k_, T89_k_, CN89_k_*) in the active pair (M3) are determined. Their variations are graphically shown in Figure 8b,c.

First, when approaching the RTRR (9, 8, 7) active modular group, it is necessary to evaluate the equivalent torques of the external and inertia forces in each link center of mass.

**Figure 9:**
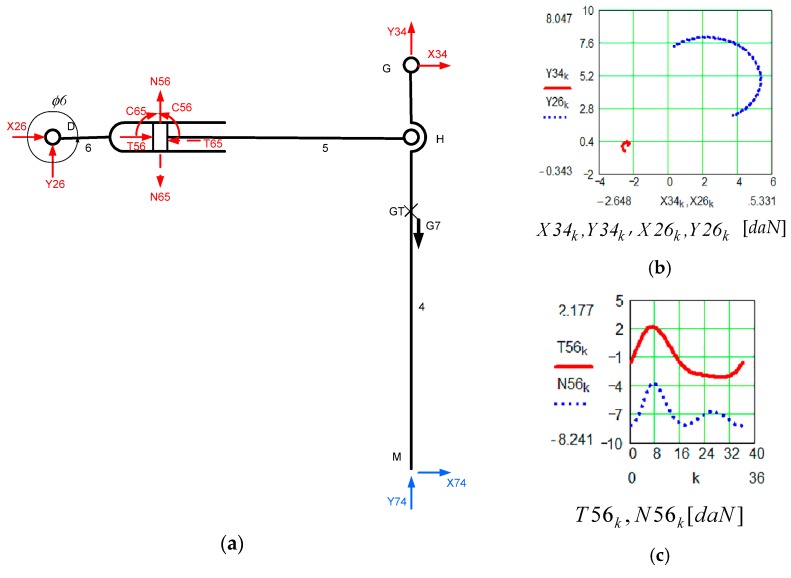
Active modular group RTRR (6, 5, 4); (**a**) reaction torque in each pair, (**b**) reaction forces variation in the rotation pairs; (**c**) reaction forces variation in the active prismatic pair.

Due to the specific construction of the mechanism previously mentioned, the only torque is defined as τ7(*X7, Y7, CM7*), which is placed in *GL*≡*GML*, the foot’s (link 7) center of mass. The active modular group RTRR (6, 5, 4) from Figure 9a is the next group analyzed and identified in the group connection (Figure 2b). This group is dedicated to the direct model of the mechanism. Following the algorithm from Table 5, it is determined which equivalent torque of the external and inertia forces for link 4 are applied in its GT center of mass (τ4 (*X4_k_, Y4_k_, CM4_k_*)). The reaction torque components in kinematic pairs are successively established and named *(X34_k_, Y34_k_)* in pair *G*, *(X26_k_, Y26_k_)* in pair *D*, *(X54_k_, Y54_k_)* in pair *H,* and *(N56_k_, T56_k_, CN56_k_)* in active pair *M*2. The variations of some significant dynamic parameters are shown in Figure 9b,c.

**Figure 10:**
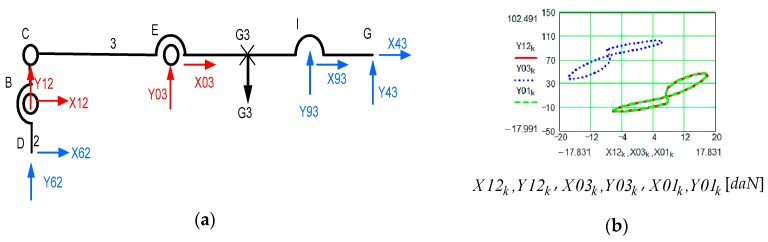
Passive modular group RRR (2, 3): (**a**) reaction torque in each pair, (**b**) reaction forces in the rotation pairs.

Next, the modular group RRR (2, 3) (Figure 10a) is analyzed (Table 5). Initially, it is necessary to calculate the equivalent torque of each link in its center of mass, which is τ2 (*X2_k_, Y2_k_, CM2_k_*) in *G2* and τ3 (*X3_k_, Y3_k_, CM3_k_*) in *G3*. The reaction torques are *(X12_k_, Y12_k_)* and *(X03_k_, Y03_k_),* respectively, in the B and E external pairs. Their variations are given in Figure 10b.

**Figure 11:**
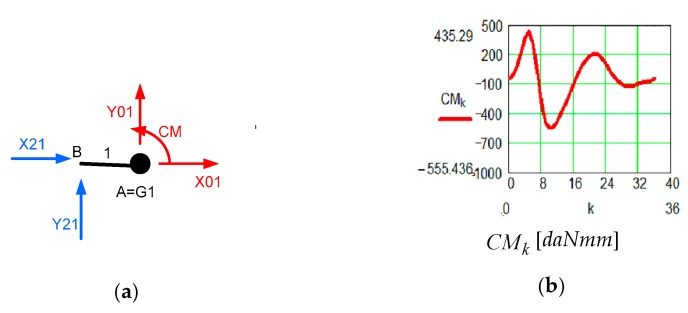
Active modular initial group AMIG (A, 1): (**a**) Reaction torque in pair A; (**b**) moment variation in the active pair A.

The final step of the dynamic analysis includes the AMIG (A, 1) modular active initial group (Figure 11a). The reaction torque in the active pair A is given by *(X01_k_, Y01_k_, CM_k_)*. Its force component variation is shown in Figure 10b. Its moment is marked *CM_k_* in Figure 11b.

## 5. Mechanism Characteristics for the Knee Joint Function Exclusive Recovery

The recovery of the knee joint and the associated muscles is accomplished on element 3 of the mechanism, wherein one flexes the medial region of the lower limb relative to the patient's seated position (Figure 2b). The angular position of link 3 is characterized by ϕ3 and is approximately placed in the (176°, 182°) interval.

**Figure 12:**
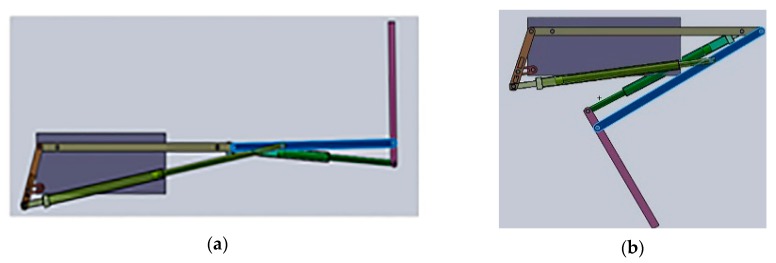
Knee joint recovery: (**a**) femur initial position with the foot maintained at 90°; (**b**) femur extreme position.

The calf solidarity with the element 4 oscillates vertically with 75° in the sagittal plane after a cycle (Figure 12), i.e., vertical, posterior, anterior, and vertical while the foot is maintained at 90°.

For this situation, *DH* and *IL* distances (Figure 2b) have to be correlated with active pairs between the elements (6, 5) and (9, 8). This should be rendered by *M*2 and *M*3 motors, while the *M*1 (active kinematic pair A) ensures the positioning of the femur in accordance with the angle of element 3.

During the cycle previously described by the kinematic element 4 (Figure 2b), the kinematic pairs *I*, *G*, and *D* maintain their immobile positions.

**Figure 13:**
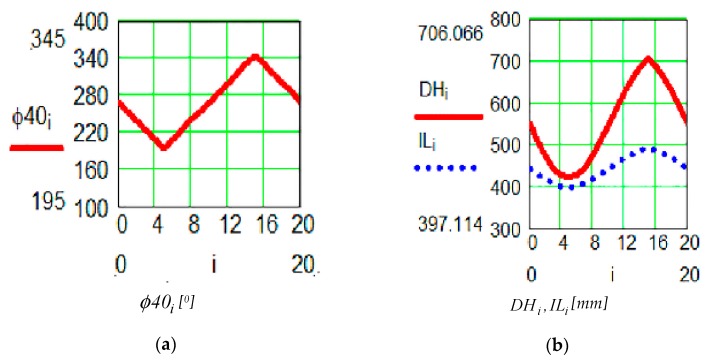
Biplet characteristics: (**a**) Angular characteristic of the link 4; (**b**) biplet linear characteristics.

The variation of the angular parameter *φ40_i_* characteristic of the leg, materialized by element 4 is shown in Figure 13a. Both biplets (6, 5) and (9, 8), as well as *DH_i_* and *IL_i_* (Figure 13b), provide mobility for link 4 and maintain the angle of 90° for the foot relative to the leg.

**Figure 14:**
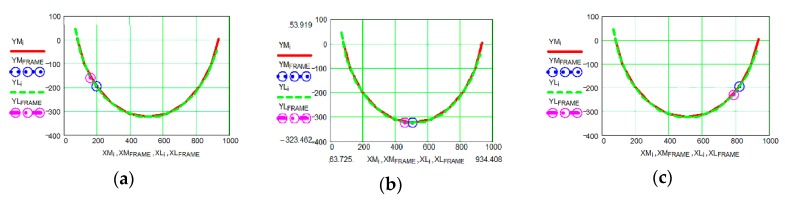
(**a**,**b**,**c**) Succession of positions of *L* and *M* points. Link 7 pairs during the recovery cycle.

Figure 14 shows the trajectories of the *L* and *M* points, as well as link 7 pairs, by which the succession of positions in the recovery cycle is checked (coordinates of pairs M, L in mm).

The reaction torque components of the kinematic pairs are determined by the following algorithm detailed in Table 5.

**Figure 15:**
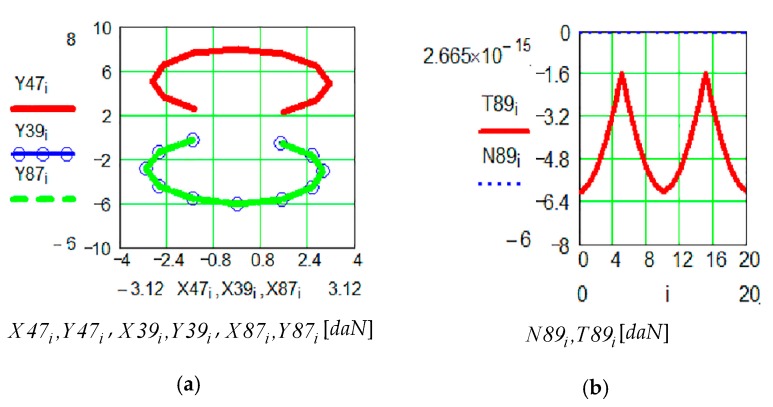
Reaction torque components for the active modular group RTRR (9, 8, 7): (**a**) Reaction force components for the rotation pairs; (**b**) reaction force components for the active pair.

From the kinetostatic analysis of the active modular group RTRR (9, 8, 7), the reactions (*X47_i_*, *Y47_i_*) for the pair *M*; (*X39_i_*, *Y39_i_*); for the pair *I*; (*X87_i_*, *Y87_i_*); for the pair *L* (Figure 15a); and the reaction torque of the active pair (8, 9) formed by the axial component *T89_i_*, the normal component *N89_i_* (Figure 15b) and the reaction moment *CN89_i_* are determined.

**Figure 16:**
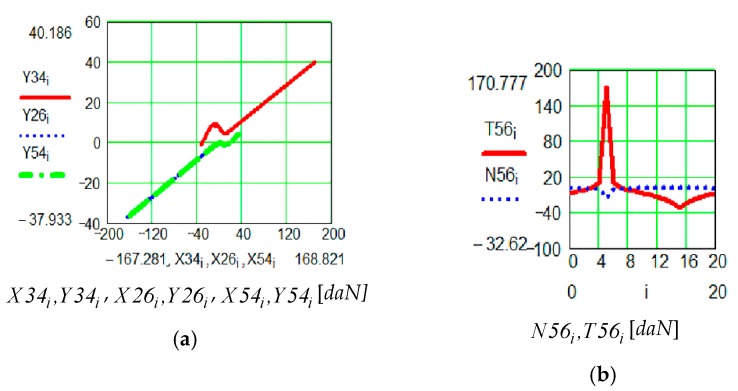
Reaction torque components for the active modular group RTRR (6, 5, 4): (**a**) Reaction force components for the rotation pairs; (**b**) reaction force components for the active pair.

The kinetostatic analysis of the modular active group RTRR (6, 5, 4), based on the algorithm in Table 5, determines the following components of the reaction torques: (*X34_i_*, *Y34_i_*) from pair *G*, (*X26_i_*, *Y26_i_*) from pair *D* (Figure 16a), and (*T56_i_*, *N56_i_*, *CN56_i_*) from the active pair (Figure 16b) between the elements (6, 5). This modular group ensures knee mobility.

**Figure 17:**
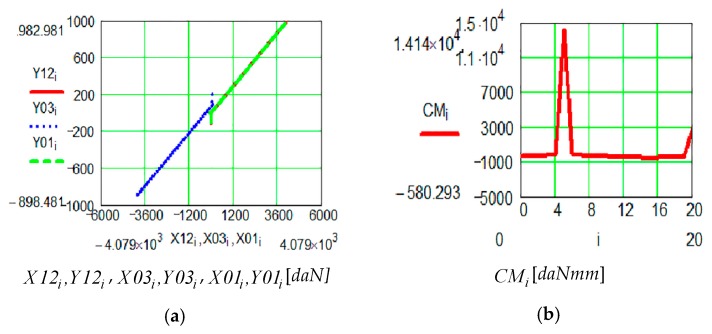
Reaction torque components: (**a**) reaction force components for the passive modular group RRR (2, 3) and active modular initial group AMIG (A, 1); (**b**) moment for the active pair A.

The kinetostatic analysis (Table 5) is continued with the passive modular group RRR (2, 3) from which the components (*X12_i_*, *Y12_i_*) in pair *B* and (*X34_i_*, *Y34_i_*) from pair *G* are determined (Figure 16a or Figure 17a).

The initial active modular AMIG (A, 1) is used to provide an instantaneous balance of the entire system. The torque components for the active pair *A* is as follows: (*X01_i_*, *Y01_i_*) (Figure 17a) and *CM_i_* (Figure 17b).

The values given in the force variation graphs are given in daN and the moment in daNmm for the geometric and mass characteristics adopted. These correspond to a standard average patient.

## 6. Mechanism Characteristics for the Talocrural Joint Exclusive Recovery

**Figure 18:**
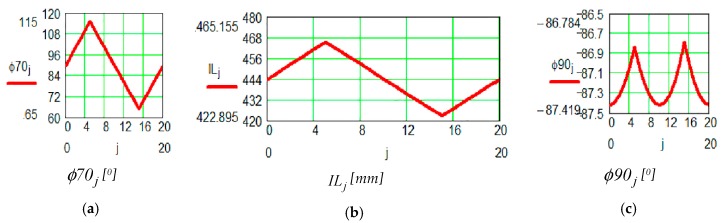
Positional characteristics of the active modular group RTRR (9, 8, 7): (**a**) Angular characteristic of link 7 (foot); (**b**) linear characteristic of the biplet (9, 8); (**c**) angular characteristic of the biplet (9, 8).

For the recovery of the talocrural joint and associated muscles, flexions of the lower limb’s extremity are performed. The patient is placed in the seated position while the leg is positioned at 90° relative to the horizontally placed femur. Consequently, the horizontal femur angle has values in the approximate interval (−4, 2.5). The leg is fixed at 90° with respect to the femur in the upright position, while the foot is initially placed at 90°. The angle *φ40* in link 4 (Figure 2b), which is fixed with the leg, has an approximate value of 272.5° in relation to the reference system. The angle *φ70* of the foot relative to the leg varies from the horizontal position as follows: 25° upwards from the horizontal position, 35° downwards below the horizontal plane, and then upwards to the horizontal position (Figure 18a).

**Figure 19:**
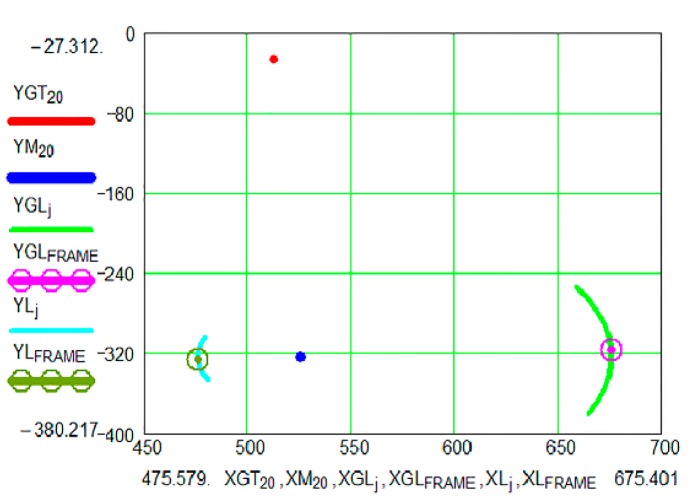
Trajectories of the various points, the materialization of the kinematic *M* and *L* pairs, and the mass centers of *GT* for the leg and *GL* for the foot.

The movement previously described is achieved through the active modular group RTRR (9, 8, 7). The variation of the linear dimension of the biplet (9, 8) for the recovery cycle is shown in Figure 18b and its angular position is *φ90_j_* (Figure 18c). The trajectories of the various points, the materialization of the kinematic *M* and *L* pairs, the centers of mass *GT* of the leg, and *GL* of the foot, emphasize the functionality of the system (Figure 19).

**Figure 20:**
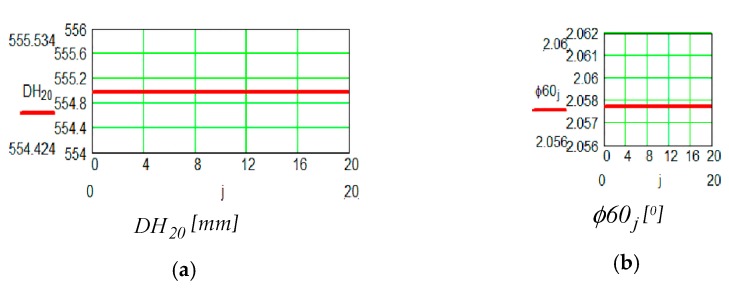
Positional characteristics of the active modular group RTRR (6, 5, 4): (**a**) linear characteristic of the biplet (6, 5); (**b**) angular characteristic of the biplet (6, 5).

The active modular group RTRR (6, 5, 4) provides the fixed position of the *G* and *D* pairs (Figure 20b). Their characteristic parameters are given by the *DH* linear parameter of the biplet (6, 5) from Figure 20a and its angle *φ60*, with respect to the fixed reference system (Figure 20b).

**Figure 21:**
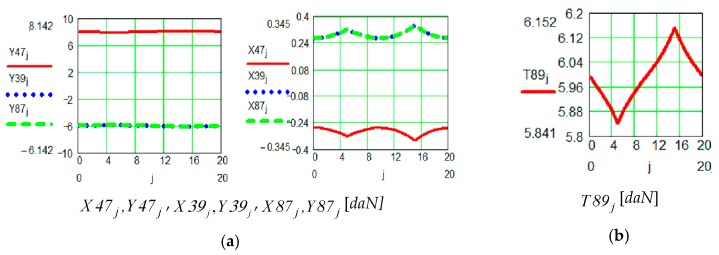
Reaction torque components for the active modular group RTRR (9, 8, 7): (**a**) Reaction force components in the rotation pairs; (**b**) force component in the active pair *M*3.

Following the algorithm presented in Table 5 for the kinetostatic analysis, the variation of the reaction torque components for the RTRR (9, 8, 7) are: (*X47_j_*, *X47_j_*) from pair *M*, (*X39_j_*, *Y39_j_*) from pair *I,* (*X87_j_*, *Y87_j_*) from pair *L* (Figure 21a), and the active component *T89_j_* (Figure 21b).

**Figure 22:**
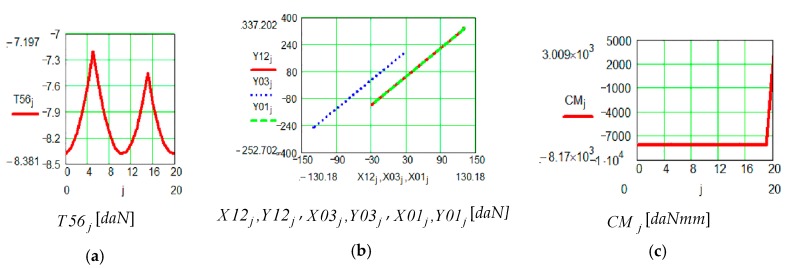
Reaction torque components: (**a**) Force component for the active pair *M*2; (**b**) reaction force components for the rotation pairs *A, B,* and *E*; (**c**) moment for the active pair *M*1-*A*.

Similarly, for the RTRR (6, 5, 4), it is assured that the immobility of the leg with respect to the femur has a reaction component that includes the active translational pair between the elements (6, 5), which further involves the axial force *T56_j_* (Figure 22a). Finally, the reactions of the *B, E,* and *A* pairs and the *CM_j_* moment of the last active modular group are shown in Figure 22b,c.

In conclusion, the data can be used for the selection of the *M*1, *M*2, and *M*3 stepping motors.

The analysis algorithm, previously mentioned for a different functional case, took into account past studies [15,16,17,18,19].

## 7. Multifunctional Medical System for Recovery and Monitoring of the Lower Limb

**Figure 23:**
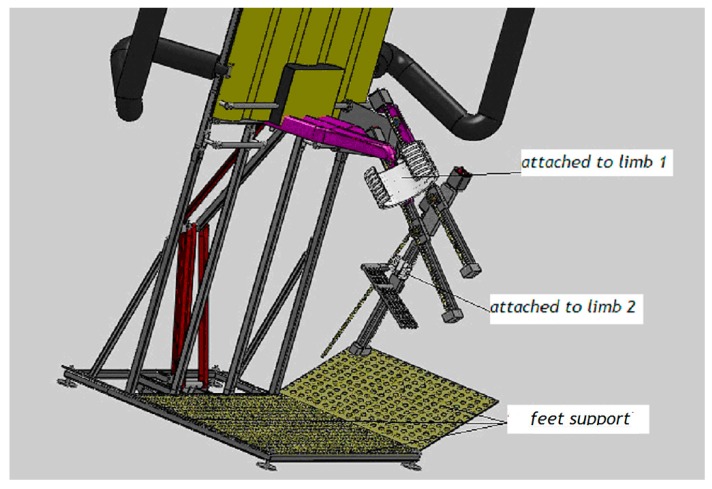
Experimental functional recovery and monitoring system.

**Figure 24:**
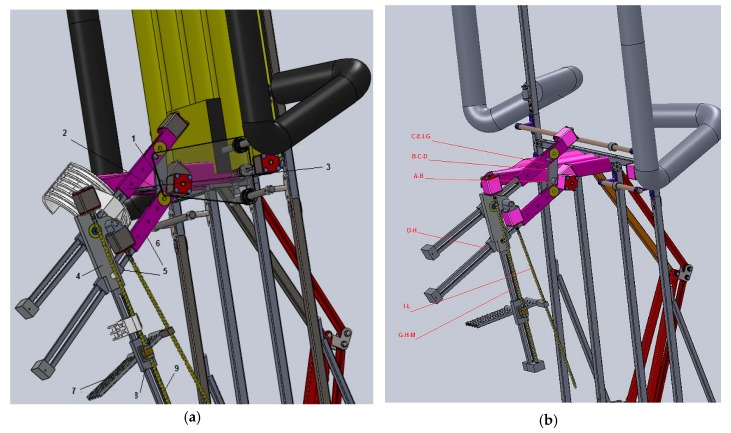
Equipment constructive details: Identification of mechanism links (**a**) and pairs (**b**) on the experimental equipment.

**Figure 25:**
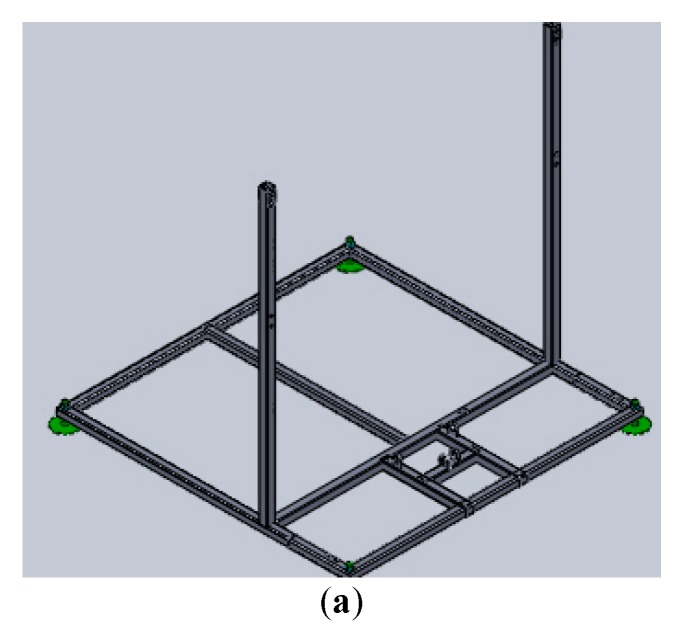

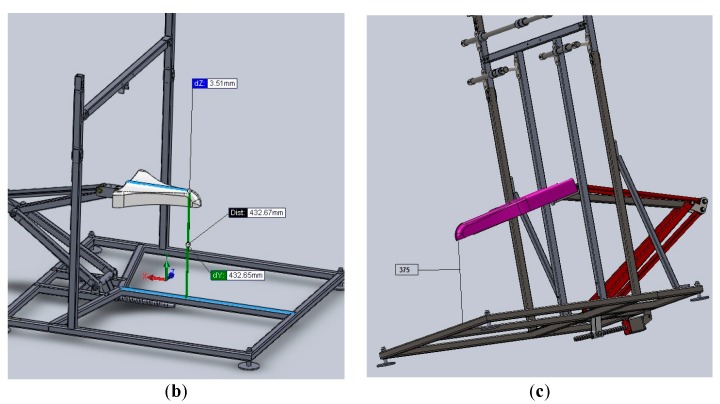
Equipment constructive details: (**a**) The medical equipment frame; (**b**) the vertical support and positioning subassembly distance at 432 mm; (**c**) the vertical support and positioning subassembly distance at 375 mm.

The medical experimental equipment (Figure 23) includes the mechanism with three degrees of mobility (Figure 2b); its kinematic links and pairs may be identified in Figure 24. On the frame (Figure 25a), the vertical support and positioning subassembly (Figure 25b) of the patient is mounted to the recovery equipment. The patient support and vertical positioning assembly of the recovery equipment ensures that each leg is placed on the corresponding recovery subassembly, while at the same time achieving the transverse adjustment of both recovery subassemblies.

**Figure 26:**
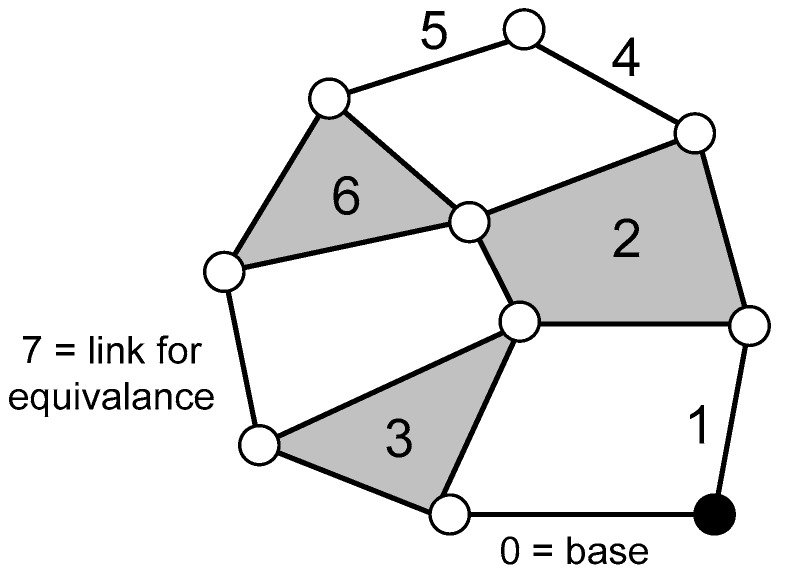
Patient positioning subassembly structural model.

The vertical support and patient positioning subassembly are based on the structural model given in Figure 26. Because the degree of mobility is unitary, the motion is achieved by the active pair between element 1 and base 0 using a stepper motor. The patient is placed on link 5. Element 7 and its adjacent pairs with elements 6 and 3 is equivalent to a superior kinematic pair materialized by a gear joint between link 3 and a geared wheel fixed on link 6.

The recovery and/or drive subassembly (Figure 24) that corresponds to each lower limb is built on the basis of a three-degree mobility mechanism (Figure 2b). The motion is provided by three motors: *M*1, *M*2, and *M*3. The three motors can work concurrently or separately depending on the necessities for recovery/training of the patient's lower limb. The segments are engaged as follows: the femur is engaged by element 3, the median region by means of element 4, and the foot by element 7.

The solutions chosen for active prismatic pairs (translation actuators) *M*2 and *M*3 are ball screw and nut mechanisms with stepping motors (Figure 1 and Figure 2b). Two such recovery and/or drive assemblies for each member can be fitted on the equipment. There were several cases where the mechanism was used for different medical applications. Taking into account the anatomic characteristics of an average person and the algorithms previously presented in the paper, the main values of the kinematic active pairs *M*1, *M*2, and *M*3 (positional and dynamic characteristics) are useful to select the step motors. These cases and values are synthesized in Table 6, Table 7 and Table 8.

## 8. Discussion

The planar or spatial mechanisms with several degrees of mobility are generally applied in robotics and often have open chains named serial ones. The mechanism created and involved in the construction of the proposed medical recovery and monitoring system is complex with closed chains. The direct and inverse structural models for multi-mobile mechanisms are useful in their kinematic and dynamic modeling. Our previous research aspired to determine the structural characteristics useful in experimental medical device design.

We ascertained that the medical device must have variable angular amplitude of each limb segment. In the future, sensors should be optimally included in the structure to ensure flexibility of the recovery and monitoring.

The equipment designed for an experimental model based on a multi-loop and multi-mobile structure can be used in different situations, namely for training each member separately or for both members for various or all joints. For such equipment, a mechanism with an open serial structure [20] is inadequate. The mechanism with three degrees of mobility and closed chains integrated in the equipment ensures flexibility in its use for recovering muscular and joint functionality in the lower limbs.

The results presented as diagrams certify the kinematic and dynamic calculation algorithm on the basis of the relations specific to the structural models derived from the classical theory of the mechanism science.

## 9. Conclusions

In this paper, we theoretically contribute to the concept of a mechanism with three degrees of mobility, which simulates the main characteristics of the human lower limb in the sagittal plane. Connected to the leg, the possibility of simultaneous or separate movement of various leg segments with variable angular amplitude is considered.

We developed the experimental equipment with students from POLITEHNICA University of Bucharest in the master’s program of Modeling and Simulation of Mechanical Mobile Systems. We included two such mechanisms, one for each leg and with the possibility of transverse adjustment. The patient was vertically placed on an adjustable chair. The adopted solution was originally distinguished from that existent in the literature.

## Figures and Tables

**Table 1:** Baranov trusses and passive modular groups.

Baranov Truss (BT) System with Three Degree of Freedom and Zero Degree of Mobility	Passive Modular Group (PMG) System with Zero Degree of Mobility	
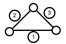 BT 1	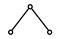	PMG 1
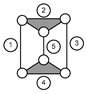 BT 2	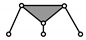	PMG 2
	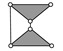	PMG 3
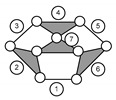 BT 3	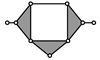	PMG 5
	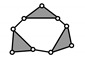	PMG 9
	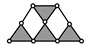	PMG 11
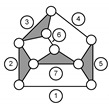 BT 4	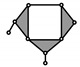	PMG 6
	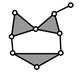	PMG 8
	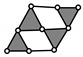	PMG 10
	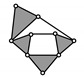	PMG 12
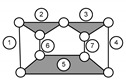 BT 5	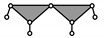	PMG 4
	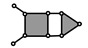	PMG 7
	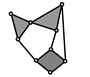	PMG 13

**Table 2:** Modular groups with one degree of mobility.

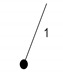	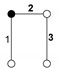	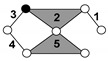	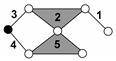
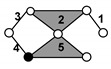	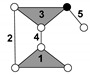	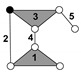	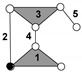

**Table 3:** Geometrical constant parameters in Figure 2 (mm).

Coordinates of the A and E Pairs Relative to the Fixed Reference System	*A(0,0),E(50,100)*
AB	30
BC	115
BD	42.39
CE	40
EG	456
EI	456–50
EG3 G3—mass center of the 3 link—femur	152
MG	444
GGT GT—mass center of the 4 link—leg	444–296
GH	151
LM	50
MGL GL—mass center of the 7 link—foot	150


**Table 4:** Mechanism positional modeling algorithm.

Dependent Parameters (Figure 2)	Positional Dependent Parameters Determination Algorithm
*B (XB, YB)* kinematic pair B coordinates	$XB_{k}=AB\cos(\phi 1_{k})$ $YB_{k}=AB\sin(\phi 1_{k})$
	$\phi 1_{k}-succesive\;angular\;position\;for\;the\;independent\;parameter\;of\;the\;A\;active\;pair$
ϕ2,ϕ3 angular parameters for PMG (2,3)–RRR (2,3)	$YB_{k}+BC\cos(\phi 2)=XE+CE\cos(\phi 3)$ $YB_{k}+BC\sin\phi 2)=XE+CE\sin(\phi 3)$ ϕ2, ϕ3-*values in rad.,* ϕ20,ϕ30*-values in ^0^.*
*C (XC, YC)* kinematic pair C coordinates	$XC_{k}=XE+CE\cos(\phi 3_{k})$ $YC_{k}=YE+CE\sin(\phi 3_{k})$
*D (XD, YD)* kinematic pair D coordinates	$XD_{k}=XB+BD\cos(\phi 2_{k}+\pi )$ $YD_{k}=YB+BD\sin(\phi 2_{k}+\pi )$
*I (XI, YI)* kinematic pair I coordinates	$XI_{k}=XE+EI\cos(\phi 3_{k}+\pi )$ $YI_{k}=YE+EI\sin(\phi 3_{k}+\pi )$
*G (XG, YG)* kinematic pair G coordinates	$XG_{k}=XE+EG\cos(\phi 3_{k}+\pi )$ $YG_{k}=YE+EG\sin(\phi 3_{k}+\pi )$
*M (XM, YM)* kinematic pair M coordinates	$XM_{k}=XG_{k}+MG\cos(\phi 4_{k})$ $YM_{k}=YG_{k}+MG\sin(\phi 4_{k})$
	$\phi 4_{k}=\phi 3_{k}+\pi -\pi /2$
*H (XH, YH)* kinematic pair H coordinates	$XH_{k}=XG_{k}+GH\cos(\phi 4_{k})$ $YH_{k}=YG_{k}+GH\sin(\phi 4_{k})$
*L (XL, YL)* kinematic pair L coordinates	$XL_{k}=XM_{k}+LM\cos(\phi 4_{k}-\pi /2)$ $YL_{k}=YM_{k}+LM\sin(\phi 4_{k}-\pi /2)$
DH,ϕ6 parameters for PMG (6, 5) = RTR (6, 5) *DH*–linear parameter, ϕ6-angular parameter	$DH_{k}=\sqrt{{\left(XH_{k}{-\mathrm{XD}}_{k}\right)}^{2}+\;{\left(YH_{k}{-\mathrm{YD}}_{k}\right)}^{2}}$
	$\tau_{k}=a\tan(\frac{YH_{k}{\;-\;\mathrm{YD}}_{k}}{XH_{k}{\;-\;\mathrm{XD}}_{k}})$
	*ϕ6_k_* = *τ_k_*, *ϕ60_k_*- *values in ^0^ of the* *ϕ6_k_ in rad*.
IL,ϕ9 parameters for PMG (9, 8) = RTR (9, 8) *IL*–linear parameter, ϕ9-angular parameter	$IL_{k}=\sqrt{{\left(XL_{k}{-XL}_{k}\right)}^{2}+\;{\left(YL_{k}{-YI}_{k}\right)}^{2}}$
	$\phi 9_{k}=a\tan(\frac{YL_{k}{\;-\;\mathrm{YI}}_{k}}{XL_{k}{\;-\;\mathrm{XI}}_{k}})$
	$\phi 9_{k}=if(\phi 9_{k}\geq 0,\phi 9_{k}+\pi ,2\pi +\phi 9_{k})$
	$\phi 90_{k}$-*values in ^0^ of the* $\phi 9_{k}$*in rad*.
*G3 (XG3, YG3)* coordinates of the 3 link-femur center of mass G3	$\begin{array}{l} XG3_{k}=XE\;+\;\mathrm{EG}3\;\cos\;(\phi 3_{k}-\;\pi )\\ YG3_{k}=YE\;+\;\mathrm{EG}3\;\sin\;(\phi 3_{k}-\;\pi ) \end{array}$
	*G3–the 3 link–femur centre of mass.*
*GT (XGT, YGT)* coordinates of the 4 link-leg center of mass GT	$\begin{array}{l} XGT_{k}=XG_{k}\;+\;\mathrm{GGT}\;\cos\;\phi 4_{k}\\ YGT_{k}=YG_{k}\;+\;\mathrm{GGT}\;\sin\;\phi 4_{k} \end{array}$
	*GT–the 4 link–leg centre of mass.*
*GL (XGL, YGL)* coordinates of the 7 link-foot center of mass GL	$XGL_{k}=XM_{k}+MGL\cos(\phi 4_{k}+\pi /2)$ $YGL_{k}=YM_{k}+MGL\sin(\phi 4_{k}+\pi /2)$
	*GL–the 7 link–foot centre of mass.*
	

**Table 5:** Algorithm for dynamic parameters.

RTRR (9, 8, 7)	The equivalent torque of the external and inertial forces for link 7 vidi Figure 8	τ7=(X7,Y7,CM7)	*X7 = 0* *Y7 = −G7* *CM7 = 0*
			G7-link 7 weight-Mass and moment of inertia for links 9 and 8 are neglected
	The reaction torque in pair M vidi Figure 8 $X47_{k}=-X74_{k}$; $Y47_{k}=-Y74_{k}$;	$X47_{k},Y47_{k}$	$A_{k}\left|\begin{array}{c} X47_{k}\\ Y47_{k} \end{array}\right|=B_{k}$
			$A_{k}=\left|\begin{array}{cc} {-(\mathrm{YM}}_{k}{-\mathrm{YL}}_{k}) & XM_{k}{-\mathrm{XL}}_{k}\\ {-(\mathrm{YM}}_{k}{-\mathrm{YI}}_{k}) & {\mathrm{XM}}_{k}{-\mathrm{XI}}_{k} \end{array}\right|$
			$B_{k}=\left|\begin{array}{c} -\left[{\mathrm{CM}7-(\mathrm{YGL}}_{k}-YL_{k})X7+{(\mathrm{XGL}}_{k}-XL_{k})Y7\right]\\ -\left[{\mathrm{CM}7-(\mathrm{YGL}}_{k}-YI_{k})X7+{(\mathrm{XGL}}_{k}-XI_{k})Y7\right] \end{array}\right|$
	The reaction torque in pair I vidi Figure 8 $X39_{k}=-X93_{k}$; $Y39_{k}=-Y93_{k}$;	$X39_{k},Y39_{k}$	$\begin{array}{l} X39_{k}=-{(X47}_{k}+X7)\\ Y39_{k}=-{(Y47}_{k}+Y7) \end{array}$
	The reaction torque in pair L vidi Figure 8	$X87_{k}=-{X78}_{k}$ $Y87_{k}=-{Y78}_{k}$	$\begin{array}{l} X87_{k}=-(X47_{k}+X7)\\ Y87_{k}=-(Y47_{k}+Y7) \end{array}$
	The reaction torque in active pair M3 vidi Figure 8	$N89_{k}$ $T89_{k}$ $CN89_{k}$	$\begin{array}{l} N89_{k}=-{(-X39}_{k}\;\sin(\phi 9_{k})+Y39_{k}\;\cos(\phi 9_{k}))\\ T89_{k}=-{(X39}_{k}\;\cos(\phi 9_{k})+Y39_{k}\;\sin(\phi 9_{k})) \end{array}$
RTRR (6, 5, 4)	The equivalent torque of the external and inertial forces for link 4	$\tau 4=(X4_{k},Y4_{k},CM4_{k})$	$\begin{array}{l} X4_{k}={-X47}_{k}\\ Y4_{k}=-{Y47}_{k}\\ CM4_{k}=(YM_{k}{-\mathrm{YGT}}_{k})X47_{k}-(XM_{k}{-\mathrm{XGT}}_{k})Y47_{k} \end{array}$
	The reaction torque in pair G vidi Figure 9	$X34_{k},Y34_{k}$	$A_{k}\left|\begin{array}{c} X34_{k}\\ Y34_{k} \end{array}\right|=B_{k}$
			$A_{k}=\left|\begin{array}{cc} {-(\mathrm{YG}}_{k}{-\mathrm{YH}}_{k}) & XG_{k}{-\mathrm{XH}}_{k}\\ {-(\mathrm{YG}}_{k}{-\mathrm{YD}}_{k}) & {\mathrm{XG}}_{k}{-\mathrm{XD}}_{k} \end{array}\right|$
			$B_{k}=\left|\begin{array}{c} -\left[{\mathrm{CM}4-(\mathrm{YGL}}_{k}-YH_{k})X4_{k}+{(\mathrm{XGL}}_{k}-XH_{k})Y4_{k}\right]\\ -\left[{\mathrm{CM}4-(\mathrm{YGL}}_{k}-YD_{k})X4_{k}+{(\mathrm{XGL}}_{k}-XD_{k})Y4_{k}\right] \end{array}\right|$
	The reaction torque in pair D vidi Figure 9	$X26_{k},Y26_{k}$	$\begin{array}{l} X26_{k}=-{(X34}_{k}+X4_{k})\\ Y26_{k}=-{(Y34}_{k}+Y4_{k}) \end{array}$
	The reaction torque in pair H vidi Figure 9	$X54_{k},Y54_{k}$	$\begin{array}{l} X54_{k}=-{(X34}_{k}+X4_{k})\\ Y54_{k}=-{(Y34}_{k}+Y4_{k}) \end{array}$
	The reaction torque in active pair *M*2 vidi Figure 9	$N56_{k}$ $T56_{k}$ $CN56_{k}$	$\begin{array}{l} N56_{k}=-{(-X26}_{k}\;\sin(\phi 6_{k})+Y26_{k}\;\cos(\phi 6_{k}))\\ T89_{k}=-{(X26}_{k}\;\cos(\phi 6_{k})+Y26_{k}\;\sin(\phi 6_{k})) \end{array}$
RRR (2, 3)	G2 center of mass coordinates vidi Figure 10	XG2,YG2	$BG2=\frac{BD+BC}{2}$
			$\begin{array}{l} XG2_{k}=XB_{k}+BG2\;\cos(\phi 2_{k})\\ YG2_{k}=YB_{k}+BG2\;\sin(\phi 2_{k}) \end{array}$
	The equivalent torque of the external and inertial forces for link 2 vidi Figure 10	$\tau 2=(X2_{k},Y2_{k},CM2_{k})$	$\begin{array}{l} X2_{k}=-{X26}_{k}\\ Y2_{k}=-{Y26}_{k}\\ CM2_{k}=(YD_{k}-YG2_{k})X26_{k}{-(\mathrm{XD}}_{k}{-\mathrm{XG}2}_{k})Y26_{k} \end{array}$
	The equivalent torque of the external and inertial forces for link 3 vidi Figure 10	$\tau 3=(X3_{k},Y3_{k},CM3_{k})$	$\begin{array}{l} X3_{k}={-X39}_{k}{-X34}_{k}\\ Y3_{k}=-{Y39}_{k}{-Y34}_{k}-G3\\ CM3_{k}=(YI_{k}{-\mathrm{YG}3}_{k})X39_{k}+(YG_{k}{-\mathrm{YG}3}_{k})X34_{k}-\\ {\;-(\mathrm{XI}}_{k}{-\mathrm{XG}3}_{k})Y39_{k}{-(\mathrm{XG}}_{k}{-\mathrm{XG}3}_{k})Y34_{k}-\\ {\;-(\mathrm{XE}-\mathrm{XG}3}_{k})G3 \end{array}$
			G3-link 3 weight
	The reaction torque in pair B vidi Figure 10	$X12_{k},Y12_{k}$	$A_{k}\left|\begin{array}{c} X12_{k}\\ Y12_{k} \end{array}\right|=B_{k}$
			$A_{k}=\left|\begin{array}{cc} {-(\mathrm{YB}}_{k}{-\mathrm{YC}}_{k}) & XB_{k}{-\mathrm{XC}}_{k}\\ {-(\mathrm{YB}}_{k}-\mathrm{YE}) & {\mathrm{XB}}_{k}-\mathrm{XE} \end{array}\right|$
			$B_{k}=\left|\begin{array}{c} {-(\mathrm{CM}2}_{k}{-(\mathrm{YG}2}_{k}{-\mathrm{YC}}_{k})X2_{k}+(XG2_{k}{-\mathrm{XC}}_{k})Y2_{k})\\ \begin{array}{l} {-(\mathrm{CM}2}_{k}{-(\mathrm{YG}2}_{k}-\mathrm{YE})X2_{k}+(XG2_{k}-\mathrm{XE})Y2_{k})+\\ +CM3_{k}{-(\mathrm{YG}3}_{k}{-\mathrm{YE})X3}_{k}+(XG3_{k}{-\mathrm{YE})Y3}_{k}) \end{array} \end{array}\right|$
	The reaction torque in pair E vidi Figure 10	$X03_{k},Y03_{k}$	$\begin{array}{l} X03_{k}=-{(X12}_{k}+X2_{k}+X3_{k})\\ Y03_{k}=-{(Y12}_{k}+Y2_{k}+Y3_{k}) \end{array}$
AMIG (A, 1)	The reaction torque in active pair A vidi Figure 11	$X01_{k}$, $Y01_{k}$, $CM_{k}$	$\begin{array}{l} X01_{k}=X12_{k}\\ Y01_{k}=Y12_{k}\\ CM_{k}=-{[(\mathrm{YB}}_{k}{-\mathrm{YA})X12}_{k}{-(\mathrm{XB}}_{k}{-\mathrm{XA})Y12}_{k}] \end{array}$

**Table 6:** Training of the femoral region and the maintenance in fixed angular positions of the calf and foot in relation to it.

***M*1 Active Pair**		
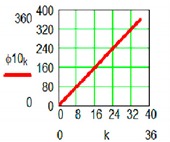	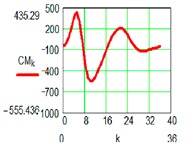	ΔCM=max(CM)−min(CM) M1 step motor moment=ΔCM/2 $\begin{array}{l} M1\;step\;\mathrm{motor}\;\mathrm{moment}=\\ \;485\;\mathrm{daN}\;\mathrm{mm} \end{array}$
Positional characteristic *$\phi 10_{k}$[^0^]*	Dynamic characteristic $CM_{k}$[*daNmm*]	
***M*2 Active Pair**		
‘ 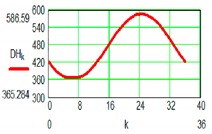	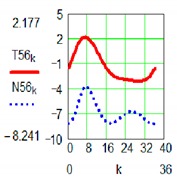	ΔT56=max(T56)−min(T56) M2 step motor axial force=ΔT56/2 $\begin{array}{l} M2\;step\;\mathrm{motor}\;\mathrm{axial}\;\mathrm{force}=\\ \;2658\;\mathrm{daN} \end{array}$
Positional characteristic $DH_{k}$*[mm]*	Dynamic characteristic $N56_{k},T56_{k}$[*daN*]	
***M*3 Active Pair**		
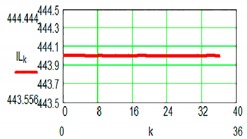	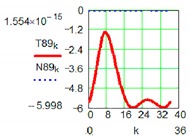	ΔT89=max(T89)−min(T89) M3 step motor axial force=ΔT89/2 $\begin{array}{l} M3\;step\;\mathrm{motor}\;\mathrm{axial}\;\mathrm{force}=\\ \;2329\;\mathrm{daN} \end{array}$
Positional characteristic $IL_{k}$*[mm]*	Dynamic characteristic $N89_{k},T89_{k}$[*daN*]	

**Table 7:** Exclusive training of the knee joint and median region.

***M*1 Active Pair**		
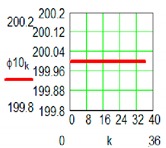	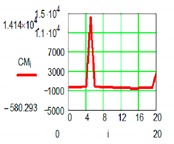	ΔCM=max(CM)−min(CM) M1 step motor moment=ΔCM/2 $\begin{array}{l} M1\;step\;\mathrm{motor}\;\mathrm{moment}=\\ \;7372\;\mathrm{daN}\;\mathrm{mm} \end{array}$
Positional characteristic *$\phi 10_{k}$[^0^]*	Dynamic characteristic $CM_{i}$[*daNmm*]	
***M*2 Active Pair**		
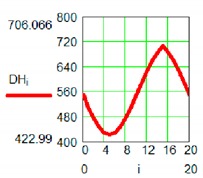	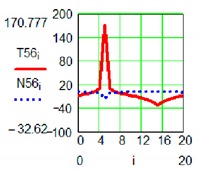	ΔT56=max(T56)−min(T56) M2 step motor axial force=ΔT56/2 $\begin{array}{l} M2\;step\;\mathrm{motor}\;\mathrm{axial}\;\mathrm{force}=\\ \;101699\;\mathrm{daN} \end{array}$
Positional characteristic $DH_{i}$*[mm]*	Dynamic characteristic $N56_{i},T56_{i}$[*daN*]	
***M*3 Active Pair**		
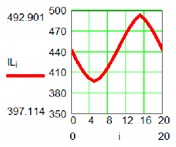	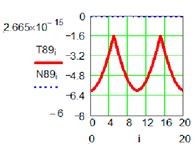	ΔT89=max(T89)−min(T89) M3 step motor axial force=ΔT89/2 $\begin{array}{l} M3\;step\;\mathrm{motor}\;\mathrm{axial}\;\mathrm{force}=\\ \;2222\;\mathrm{daN} \end{array}$
Positional characteristic $IL_{i}$*[mm]*	Dynamic characteristic $N89_{i},T89_{i}$[*daN*]	

**Table 8:** Exclusive training of the talocrural joint (ankle).

***M*1 Active Pair**		
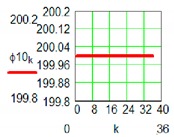	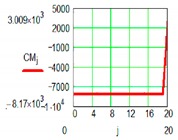	ΔCM=max(CM)−min(CM) M1 step motor moment=ΔCM/2 $\begin{array}{l} M1\;step\;\mathrm{motor}\;\mathrm{moment}=\\ \;5590\;\mathrm{daN}\;\mathrm{mm} \end{array}$
Positional characteristic *$\phi 10_{k}$[^0^]*	Dynamic characteristic $CM_{j}[daNmm]$	
***M*2 Active Pair**		
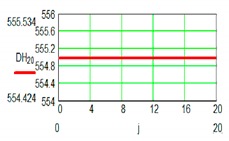	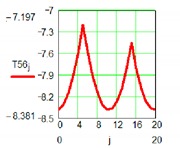	ΔT56=max(T56)−min(T56) M2 step motor axial force=ΔT56/2 $\begin{array}{l} M2\;step\;\mathrm{motor}\;\mathrm{axial}\;\mathrm{force}=\\ \;0.492\;\mathrm{daN} \end{array}$
Positional characteristic $DH_{20}[mm]$	Dynamic characteristic $T56_{j}[daN]$	
***M*3 Active Pair**		
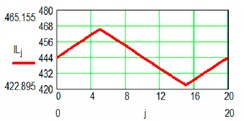	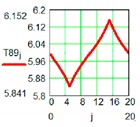	ΔT89=max(T89)−min(T89) M3 step motor axial force=ΔT89/2 $\begin{array}{l} M3\;step\;\mathrm{motor}\;\mathrm{axial}\;\mathrm{force}=\\ \;0.156\;\mathrm{daN} \end{array}$
Positional characteristic $IL_{j}[mm]$	Dynamic characteristic $T89_{j}[daN]$	
